# Morphological and Prognostic Values of Skin Lesions in Patients with COVID-19

**DOI:** 10.1155/2024/4975523

**Published:** 2024-10-22

**Authors:** Mahmoud A. Rageh, Ibrahim H. E. Yousef, Yaser Hosny Ali Elewa, Mofreh Mansour, Omar AbdelHady Omar Ahmed, Sameh Fawzy Fahmy, Ahmad Saeed Aladl, Mohamed Amer, Emad El Rewiny, Manar Elsayed Sallam, Amr Mohammad Ammar, Salma S. Mohammed, Ahmed Shawky

**Affiliations:** ^1^Department of Dermatology, Faculty of Medicine, Al-Azhar University, Cairo, Egypt; ^2^Chest Diseases Department, Faculty of Medicine, Al-Azhar University, Cairo, Egypt; ^3^Department of Histology and Cytology, Faculty of Veterinary Medicine, Zagazig University, Zagazig, Egypt; ^4^Department of Basic Veterinary Sciences, Faculty of Veterinary Medicine, Hokkaido University, Hokkaido 060-0818, Japan; ^5^Department of Dermatology, Faculty of Medicine for Girls, Al-Azhar University, Damietta, Egypt; ^6^Department of Dermatology, Andrology and STDs, Faculty of Medicine, Mansoura University, Mansoura, Egypt; ^7^Department of Dermatology, Alzahra Hospital, Alazhar University, Cairo, Egypt

## Abstract

The dermatological manifestations of the coronavirus cause severe acute respiratory syndrome. The current study investigates the morphological and histopathological relationship between the emergence of skin lesions and the severity of COVID-19 across the course of the disease via a cross-sectional study. There were skin lesions (maculopapular rash, vesiculobullous lesions, urticarial lesions, cutaneous thromboembolic “CT,” and erythema multiforme-like lesions “EM-like”) in confirmed COVID-19 instances. A total of 150 patients of both sexes were evaluated morphologically and were classified as early (44% of the total) or late based on the emergence of respiratory symptoms (one week before or two weeks after, respectively). The early and late diagnostic groups represented 44% and 56% of the total patients, respectively. Patients with no skin lesion and one skin lesion showed a significant correlation with disease timeline early and late stages (*X*2 = 22.38, *P* < 0.001; *X2* = 4.432, *P* < 0.001, respectively). CT and EM-like were correlated with the disease severity, *X*2 = 50.51, *P* < 0.001; *X*2 = 19.186, *P* ≤ 0.001, respectively. In conclusion, our data suggested that the onset of dermatological lesions that developed during the COVID-19 pandemic may be a useful diagnostic and prognostic tool for COVID-19 severity. Therefore, a thorough examination of the skin may save time and bring physicians to an accurate diagnosis and, as a result, prompt treatment.

## 1. Introduction

Since coronavirus disease 2019 (COVID-19) has developed and progressed into a pandemic, a significant body of literature has been accumulating handling the various clinical, diagnostic, therapeutic, and protective aspects of the pandemic, the global count of COVID-19 cases has exceeded 6,421,958, and the global death toll has spiked up to 6,52,308 from December 2019 to 27 July 2020. The virus has been labeled as a pandemic by WHO [1, 2]. The typical clinical features of COVID-19 illness are fever, cough, lack of smell, and lymphocytopenia. Despite being less prevalent, dermatological manifestations of COVID-19 are one of the clinical signs that have been recorded [3]. Dermatological manifestations, including viral exanthemas, often accompany viral infection. Infection with severe acute respiratory syndrome coronavirus-2 (SARS-CoV-2) was no exception. Consequently, the dermatological manifestations of SARS-CoV-2 infection have become the focus of COVID-19 research [4].

A population-based timeline framework was proposed for a more thorough and structured perspective of the natural history of the symptomatic SARS-CoV-2 infection [5]. Accordingly, the periods of SARS-CoV-2 viral infection have been divided into three phases elucidating the involved pathophysiological processes, the characteristic clinical features, and the dominant laboratory findings. These phases include acute infection representing the first two weeks from the onset of the disease [6, 7], the postacute hyperinflammatory illness extending between week two and week 4 [8, 9], and the late sequelae phase extending beyond the fourth week of illness [10]. According to Datta et al.'s [5] assumption, skin lesions are more likely to appear in the first four weeks of COVID-19 onset, whereas both prodromal and late-onset lesions were rare [11–13]. Therefore, it was suggested that skin manifestations could be the heralding symptoms of SARS-CoV-2 [11], albeit skin lesions cannot be given as an indicator of COVID-19 severity [13–16].

The incidence of skin lesions in patients with COVID-19 was estimated to range from 0.2% up to 20.4% [11, 12, 17], with the main lesions including erythematous-maculopapular, urticarial, papulovesicular, and purpuric-petechial eruptions, chilblain-like lesions, and livedoid-acro-ischemic lesions [18]. However, one study reported that among 1053 tracked cases, only 8 patients developed dermatological reactions potentially attributable to COVID-19: 7 episodes of inguinal and intergluteal intertrigo, and 1 case of erysipelas of the right lower limb [19]. The literature reported different skin lesions in patients with COVID-19, thus highlighting the diversity of mechanisms through which the SARS-CoV-2 virus interacts with the skin [17, 20]. Even though the skin manifestations of COVID-19 have been well characterized, the underlying cause of skin lesions is still a conundrum. There are different hypotheses explaining the SARS-CoV-2 infection of skin cells, with direct invasion of skin cells being proposed as a possibility. The spike protein of SARS-CoV-2 [21] attaches to angiotensin-converting enzyme 2 (ACE-2) receptors on the surface of keratinocytes and sweat glands [22]. This hypothesis is consistent with the fact that epidermal and adnexal necrosis was detected in all skin lesions in patients with COVID-19 [13].

Moreover, skin lesions can result from the virus-mediated immune system response with the secretion of cytokines and growth factors [23]. Furthermore, coinfection with other viruses has been emphasized as a possibility of skin lesions in patients with COVID-19 due to the moderation of the immune system. The viral coinfection hypothesis is supported by the presence of vesicular and erythematous lesions with characteristic histological appearance compared to other skin lesions usually seen with COVID-19 alone [24, 25] Varicella infection was reported in four patients with COVID-19 presented with diffuse vesicular lesions [26]. Therefore, it is remarkable that skin lesions related to SARS-CoV-2 infection can be challenging to differentiate from other infectious exanthems and dermatoses [27]. Notwithstanding, there is a growing body of literature that is expanding our understanding of the clinical aspects, pathophysiological causes, and therapeutic management of COVID-19-associated skin lesions; there needs to be more information available synchronizing the onset of skin lesions with the course of COVID-19. Consequently, this research aimed to explore the correlation between the onset of skin lesions and COVID-19 severity along the timeline course of the disease.

## 2. Materials and Methods

### 2.1. Study Design and Setting

This cross-sectional study was conducted between October 2020 and March 2021 in the Faculty of Medicine, Al-Azhar University, Cairo, Egypt.

### 2.2. Participants

Patients were enrolled in COVID-19 follow-up units of the university hospital. All patients diagnosed as confirmed cases with COVID-19 by the RT-PCR test of nasopharyngeal or throat swabs and signed informed consent were recruited into the study.

### 2.3. Study Procedure

Patients were enrolled in COVID-19 follow-up units at the university hospital. These units were specifically established to monitor and manage patients diagnosed with COVID-19, focusing on those who required ongoing medical evaluation due to the complexity or severity of their illness. Confirmed COVID-19 patients who provided signed informed consent were included in the study. All patients were subjected to complete history taking, including age, sex, duration of symptoms, chronic diseases, and received medications. Laboratory and radiological evaluations (chest X-ray, computed tomography (CT), or RT-PCR) were done for all recruited patients. Patients recruited in the study were classified according to the severity of COVID-19 infection into mild, moderate, and severe cases according to the guidelines of the Centers for Disease Control and Prevention (CDC) [28]. Mild cases are defined as patients who have signs and symptoms of COVID-19 such as fever, cough, headache, sore throat, nausea, vomiting, muscle pain, and loss of smell and taste but no other symptoms of lower respiratory tract infection, including dyspnea or shortness of breathing, and no abnormal findings on chest radiography. Moderate cases are defined as patients with, in addition to the clinical symptoms of SARS-CoV-2 infection, clinical findings or imaging suggestive of lower respiratory tract infections and oxygen saturation (SpO2) ≥94% on room air. Severe cases are identified as patients, in addition to the previous features, having SpO2 <94% on room air, respiratory rate >30 breaths per minute, or lung infiltrates more than 50% on chest CT scans [29].

Skin lesions were assessed clinically including maculopapular rash, vesiculobullous lesions, urticarial lesions, cutaneous thromboembolic manifestations, erythema multiforme (EM)-like lesions, and other rare cutaneous signs. Skin lesions that appeared within one week before the respiratory symptoms were considered early, while skin lesions that appeared within two weeks after the respiratory symptoms were considered late. The type of skin lesions and time of their appearance were correlated with the clinical severity of COVID-19.

### 2.4. Data Collection

Data collected included demographic data, the onset of COVID-19 in confirmed cases, oxygen saturation, and comorbidities including, but not limited to, diabetes mellitus, hypertension, cardiac disorders, and renal function abnormalities. In addition, the morphological features of skin lesions were recorded concerning the time of appearance.

### 2.5. Study Outcomes

  Primary outcome: The frequency of skin lesions concerning the date of onset of COVID-19.
  Secondary outcome: The severity of COVID-19 according to oxygen saturation and comorbid conditions.

### 2.6. Ethics

The study was conducted following the Declaration of Helsinki and its amendments. Informed consent was obtained from each patient participating in the study and approved by the Institutional Review Board (or Ethics Committee) of the Faculty of Medicine, Al-Azhar University Hospitals, and the head of the Dermatology, Andrology, and Venereology department (protocol code Der_med_23.00000247).

### 2.7. Statistical Analysis

The collected data were revised, coded, tabulated, and statistically analyzed using the Statistical Package for Social Science (SPSS), Version 24.0 (IBM Corp). Data were checked for normality using the Kolmogorov–Smirnov normality test. The nonparametric variables were presented with numbers and percentages. The parametric variables were presented in the mean and one standard deviation (mean ± SD). Differences were assessed using the chi-square test. Pearson's correlation and chi-square at the 0.05 level tested the correlation between the groups.

## 3. Results

### 3.1. Demographic Characteristics of the Participants

This study included 150 patients, 52% (*n* = 78) were males, and 48% (*n* = 72) were females; the difference between males and females was nonsignificant, as revealed (*χ*^2^= 0.240; *P* = 0.624). The mean age of the patients was 37.1 ± 16.1 years (11–79 years) (Supplementary Figure 1). The early diagnosis group represented 44% (*n* = 66) of the total patients; 54.5% (*n* = 36) were male, and 45.5% (*n* = 30) were female, with a mean age being 32 ± 17 years (11–75 years). The late diagnosis group included 56% (*n* = 84) of the total patients; 50% (*n* = 42) were male, and 50% (*n* = 42) were female, with a mean age being 41.1 ± 14.2 years (16–79) years. The early diagnosis group was highly significant compared with age to the late diagnosis group (*P* < 0.001^∗∗∗^), as revealed by the independent sample *t*-test. Considering the general conditions of late diagnosis, patients showed a significant result (*P* < 0.001) in bad conditions when compared with the early group (Table 1, Flow diagram 1).

### 3.2. Qualitative Analysis

**Figure 1:**
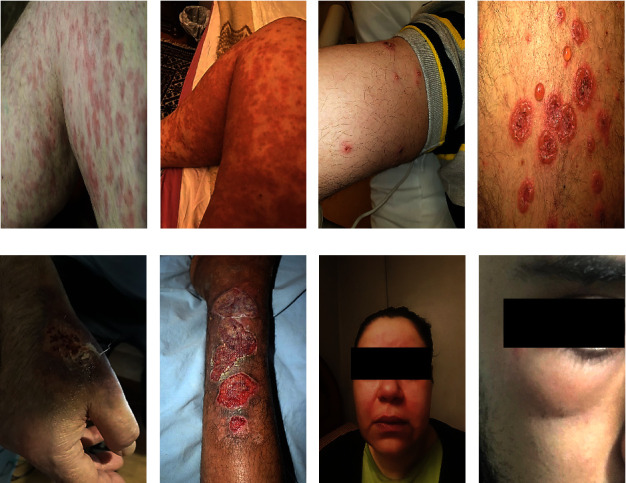
Showing early and late stages of various clinical lesion diagnoses, encompassing (a, b) maculopapular rash “(a) initial stage, (b) late stage”; (c, d) vesiculobullous lesions “(c) early emergence, (d) late stage”; (e, f) pyoderma gangrenosum “(e) early stage, (f) advanced stage”; (g, h) angioedema “(g) early stage, (h) advanced stage.” These visual representations aid in the recognition and differentiation of these clinical conditions at various stages of development.

According to the findings of the study, dermatological disorders are characterized by an extensive variety of symptoms, each of which signifies a unique pathological mechanism. The maculopapular rash (Figures 1(a) and 1(b)), characterized by the coexistence of raised papules and flat, red macules, is commonly linked to viral infections or untoward drug reactions. Vesiculobullous lesions (Figures 1(c) and 1(d)), which are distinguished by the existence of bullae or vesicles filled with fluid, are commonly observed in the context of medical conditions like pemphigus and herpes infections. Urticarial lesions appear as raised, pruritic effusions, bearing a resemblance to hives, and are frequently induced by allergic reactions. Cutaneous thromboembolic manifestations, which manifest in various diseases such as vasculitis, manifest as painful and discolored regions of the skin caused by blood vessel obstruction. Circular patterns resembling erythema multiforme are distinguished by concentric red rings that function as indicators of immune-mediated hypersensitivity reactions. These reactions are often associated with substance use or infections.

Additional lesions, such as angioedema, a condition distinguished by the sudden expansion of subcutaneous tissues, frequently observed in the periorbital and perioral areas, have been documented (Figures 1(g) and 1(h)). There are various factors that can induce this condition, including hypersensitivity reactions and the administration of specific medications. This syndrome may exhibit a correlation with urticaria and, in certain instances, may indicate a profound systemic allergic reaction. Pyoderma gangrenosum, conversely, is a rare ulcerative dermatological disorder distinguished by the rapid progression of excruciating necrosis within the skin (Figures 1(e) and 1(f)). The advancement from minor pustules to more substantial, subsided ulcers distinguished by a violet or crimson border is often linked to systemic ailments like rheumatoid arthritis or inflammatory bowel disease. Except for lesions such as angioedema and pyoderma gangrenosum, all lesions were included in the quantitative analysis due to not enough lesion counts.

### 3.3. Comorbidities

Diabetes mellitus was found in 32% (*n* = 48) of the patients, while hypertension was found in 16% (*n* = 24). There was a weak direct significant correlation between cases of diabetes mellitus and hypertension and the time of disease, COVID-19 onset (Pearson's *r* = 0.323; *P* < 0.001; CI: 0.167–0.463).

### 3.4. The Degree of Disease Severity

**Figure 2:**
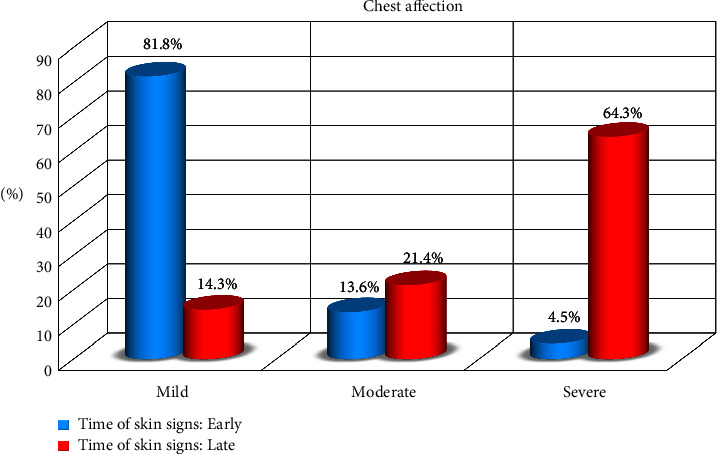
Relation between time of skin signs and chest affection.

**Figure 3:**
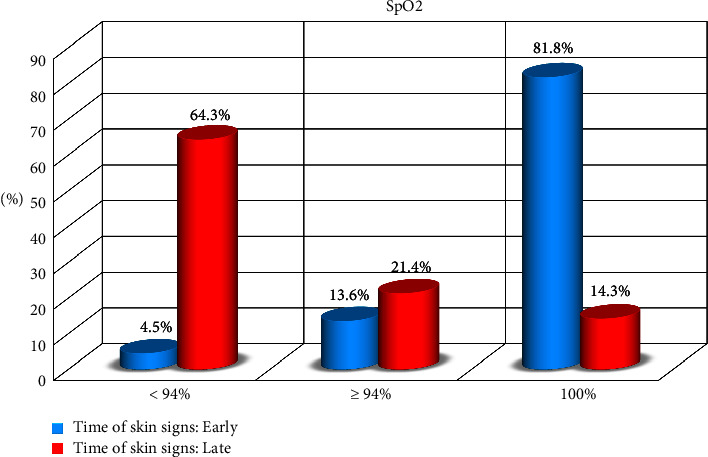
Relation between time of skin signs and peripheral oxygen saturation (SpO2).

Among COVID-19 patients, mild cases were 44% (*n* = 66), moderate cases were 18% (*n* = 27), and severe cases were 38% (*n* = 57) of all patients recruited in the study. There was a significant difference between the degree of severity and the time of disease, COVID-19, onset (*P* < 0.001). Serum oxygen partial pressure (SpO2) was 100% in 38% (*n* = 57), ≥94% in 18% (*n* = 27), and <94% in 38% (*n* = 57) of patients. There was a significant difference between SpO2 and the onset of COVID-19 disease (Table 2, Figures 2 and 3).

### 3.5. Cutaneous Manifestations

Among the recruited patients (*n* = 150), 231 skin lesions were detected and diagnosed. In the early group, 44% (*n* = 66) of the patients and 33.76% (*n* = 78) of the overall skin lesions were detected and diagnosed. In the late group, 56% (*n* = 84) of the patients and 66.32% (*n* = 153) of the overall skin lesions were detected and diagnosed (Table 3).

Regarding the number of campaigned lesions, the patients had been classified as having no lesions, one lesion, two lesions, three lesions, four lesions, and five lesions. The chi-square test showed significant correlations between the five categories against the timeline of the disease, either early appearance or late appearance (*X*^2^ = 22.38, *P*-value = 0.00044). Chi-square post hoc analysis with Bonferroni correction was performed showing significant correction for “no lesions” and “one lesion” categories only. The other categories were not significantly correlated (Table 3).

The maculopapular rash was present in 32% (*n* = 48) of all patients, 40.9% (*n* = 27) of which were presented in the early diagnosis group, and 25% (*n* = 21) were presented in the late group. There was a significant correlation between the appearance of the maculopapular rash in both groups (Table 4 and Figures 1(a) and 1(b)) (*X*^2^ = 4.299, *P*=0.038).

Vesiculobullous lesions were diagnosed in 39.3% (*n* = 59) of all patients, 45.5% (*n* = 30) in the early-diagnosed group, and 34.5% (*n* = 29) in the late-diagnosed group. There was no significant correlation between the vesiculobullous lesion in both the early and late groups (Table 4 and Figures 1(c) and 1(d)) (*X*^2^ = 1.851, *c* = 0.174).

Urticarial lesions were diagnosed in 24% (*n* = 36), 18.2% (*n* = 12) were diagnosed in the early group, and 28.6% (*n* = 24) were diagnosed in the late group. There was no significant correlation between the urticarial lesions in either the early and late groups (Table 4) (*X*^2^ = 0.121, *P*=0.079).

Cutaneous thromboembolic manifestations were diagnosed in 30% (*n* = 45) of all patients, representing 53% of the late group. No cutaneous thromboembolic lesions were detected in the early group (Table 4). (*X*^2^ = 50.51, *P* < 0.001).

Erythema multiforme-like (EM-like) lesions were diagnosed in 14% (*n* = 21). The EM-like lesions were diagnosed in the late group, representing 25% (*n* = 21) of the patients in the late group. The EM-like lesions were not detected in the early group (Table 4) (*X*^2^ = 19.186, *P* ≤ 0.001).

Other cutaneous lesions were diagnosed in 14% (*n* = 21). There was no significant correlation between the other rare lesions in either the early and late groups (Table 4) (*X*^2^ = 0.013, *P*=0.909).

## 4. Discussion

Recognizing COVID-19-related cutaneous manifestations may assist physicians in the early identification of illness before the onset of respiratory symptoms. In addition, another potential application is that cutaneous lesions may be applied to detect the clinical condition that requires therapy. According to this research, less than half of the skin lesions emerged in the early phase of the illness, whereas most skin lesions appeared in the late phase of the illness. When evaluating a patient, it is common to discover that they have multiple lesions. Patients diagnosed with COVID-19 with more than one skin lesion comprised less than one-third of the total patients. The maculopapular rash, vesiculobullous lesions, and urticaria accounted for the vast majority of the early-phase skin lesions. On the other hand, cutaneous thromboembolic manifestations and erythema multiforme-like lesions were only seen in the late phase of the COVID-19 illness, indicating serious conditions [17].

In one study, skin lesions were categorized into six dermatological clinical morphologies, including urticaria, vesicular, acral, morbilliform, petechial, and livedo reticularis, among other rare lesions [30, 31]. Another study concluded that five common skin lesions were clinical manifestations of COVID-19 more frequently in Europe and the United States than in Asia. These skin lesions included pseudo-chilblains, maculopapular, urticarial, vesicular, and vaso-occlusive lesions [17]. Chilblains-like acral patterns represented nearly half of the overall skin lesions with earlier presentation than other skin lesions in two-thirds of COVID-19 patients [4]. A chilblains-like lesion was estimated to be the most common skin lesion in Europe and the United States [17]. Furthermore, chilblain-like lesions and acro-ischemia were concluded to have a prognostic value [18]. In an international study involving 716 patients from 31 countries, the most prevalent cutaneous morphological appearances in patients with COVID-19 were morbilliform, pernio-like, urticarial, macular erythema, vesicular, papulosquamous, and retiform purpura that were correlated with the severity of illness [12].

Compared to the literature, the findings of this study emphasized that maculopapular rash, vesiculobullous lesions, and urticaria were more prevalent among patients with COVID-19 and presented early in the course of the disease. Contrary to the Western population-based literature [17], the chilblain-like acral pattern was scarce in the Egyptian population. Urticaria and maculopapular rash are globally presented with differences in prevalence. The overlapping of the morphological lesions suggests that skin lesions could be predictive diagnostic values for SARS-CoV-2 infection. Moreover, this study correlates well the appearance of a skin lesion with the onset and severity of the illness.

During the COVID-19 pandemic, skin lesions showed diverse morphological presentations, and the correlation with severity has been a matter of concern [32]. The spectrum of skin lesions spans from chilblain-like on one end to vascular lesions on the other end of the spectrum; thus, the mere occurrence of skin lesions in patients with COVID-19 may not be an indicator of disease severity [13]. On the other hand, cutaneous vaso-occlusive lesions were found to be correlated with ICU admission [17] and serious outcomes [13]. Retiform purpura was seen exclusively in critically ill, hospitalized patients [12]. Moreover, livedoid lesions were concluded to be associated with severe forms of COVID-19 [11].

Compared with the literature, the current study's findings showed that the cutaneous thromboembolism manifestations and erythema multiforme-like lesions were correlated with the late phase of COVID-19 with serious implications. Therefore, this study concurs with the suggestion that skin lesions may have prognostic value. The discrepancy in the correlation between skin lesions and the degree of severity of COVID-19 can be attributed to differences in immune reactions [33].

### 4.1. Strengths and Limitations

The main strength of this study is the correlation between skin lesions and the onset of COVID-19. Accordingly, this study highlighted the importance of skin lesions as a diagnostic and prognostic clinical feature in the COVID-19 pandemic. The second point of strength is the characterization of the dermatological features of COVID-19 in Egypt (a Middle Eastern country), emphasizing the difference from the Western dermatological features.

This study, conducted in a single academic healthcare facility, may not fully represent the dermatological features and prevalence of skin lesions in COVID-19 patients across the Egyptian population. Selection bias is possible, as patients seeking care or referred to specialized units might not reflect the entire spectrum of COVID-19 cases, and excluding those who did not provide informed consent could introduce additional bias. Retrospective data collection may lead to reporting bias due to variability in symptom reporting and comorbidity documentation. The single-center design also limits generalizability to other settings with different COVID-19 prevalence, healthcare infrastructure, and demographics. Additionally, as a cross-sectional study, it can only establish associations, not causality. Nonetheless, our study provides valuable insights into the dermatological manifestations of COVID-19 and their association with disease severity and comorbidities, supported by a robust methodology and careful consideration of potential biases. Further comprehensive studies, including multiple academic institutions and high-ranked healthcare centers, are recommended.

## 5. Conclusions

In this study, skin lesions that occurred during the COVID-19 pandemic might be an effective diagnostic and prognostic clinical tool. An exhaustive skin inspection may help save time and lead physicians to the correct diagnosis and, consequently, early treatment. Patients infected with COVID-19 often have urticaria in tandem with other symptoms of their disease. Patients diagnosed with COVID-19 almost always exhibit maculopapular rash, which is indicative of a low-to-moderate degree of disease severity. Lastly, vascular occlusive lesions are significantly related to the late stage of the disease, which is associated with a poor prognosis.

## Figures and Tables

**Table 1:** Description of demographic data and general conditions in all studied patients.

Parameters		All studied patients		Early		Late		Significance	
		*n*	%	*n*	%	*n*	%	*X* ^2^	*P* value
		150	100%	66	44.0%	84	56%	2.16	0.142ns

Age (year)	Mean ± SD	37.12 ± 16.1		32 ± 17		41.1 ± 14.2		97.24	<0.001^∗∗∗^
	Min-max	11–79		11–75		16–79			

Sex	Male	78	52.0%	36	54.5%	42	50.0%	0.306	0.580ns
	Female	72	48.0%	30	45.5%	42	50.0%		

General conditions	Good	75	50.0%	60	90.9%	15	17.9%	78.89	<0.001^∗∗∗^
	Bad	75	50.0%	6	9.1%	69	82.1%		

Note 1: *X*^2^: chi-square test. ⁣^∗^ = mildly significant, ⁣^∗∗^ = moderately significant, ⁣^∗∗∗^ = highly significant at *P* < 0.05, <0.01, <0.001, ns, nonsignificant at *P* > 0.05.

**Table 2:** Correlation between chronic diseases, chest affection, and SpO2 regarding the time of disease, COVID-19 onset.

Parameters		All studied patients		Early		Late		Correlations	
		*N*	%	*N*	%	*N*	%	*X* ^2^	*P* value
		150	100	66	44.0	84	56		

Chronic diseases	No	78	52.0	45	68.2	33	39.3	0.33	0.070ns
	DM	48	32.0	18	27.3	30	35.7		
	HTN	24	16.0	3	3.0	21	25.0		

Chest affection	Mild	66	44.0	54	82	12	14.3	74.2683	<0.001⁣^∗∗∗^
	Moderate	27	18.0	9	13.6	18	21.4		
	Severe	57	38.0	3	4.5	54	64.3		

SpO2	<94%	57	38.0	3	4.5	54	64.3	74.2683	<0.001⁣^∗∗∗^
	≥94%	27	18.0	9	13.6	18	21.4		
	100%	66	44.0	54	81.8	12	14.3		

⁣^∗^Note 2: SpO2: peripheral oxygen saturation. *X*^2^: chi-square test. *P* value >0.05 is not significant. ⁣^∗^ = mildly significant, ⁣^∗∗^ = moderately significant, ⁣^∗∗∗^ = highly significant.

**Table 3:** Correlation between the number of lesions detected regarding the time of disease, COVID-19, and onset.

Parameters		All studied patients		Early		Late		Correlations		
		*N*	Percent (%)	*N*	Percent (%)	*N*	Percent (%)	*X* ^2^	*P* value	Significance
		150	100	66	44.0	84	56	22.38⁣^∗^	0.00044	Significant

Total numbers of campaigned different lesions	No lesion	93	62.0	54	81.8	39	4.360	4.432⁣^∗∗^	<0.001	Significant
	One lesion	45	30.0	12	18.2	33	39.3	−2.799⁣^∗∗^	0.614	Significant
	Two lesion	3	2.0	0	0.0	3	3.6	−1.550⁣^∗∗^	1	Nonsignificant
	Three lesion	6	4.0	0	0.0	6	7.1	−2.216⁣^∗∗^	0.320	Nonsignificant
	Four lesion	2	1.3	0	0.0	2	2.4	1.262⁣^∗∗^	1	Nonsignificant
	Five lesion	1	0.7	0	0.0	1	1.2	−0.889⁣^∗∗^	1	Nonsignificant

The chi-square test of all categorical groups. Chi-square post hoc test with Bonferroni correction. ⁣^∗^ = mildly significant, ⁣^∗∗^ = moderately significant, ⁣^∗∗∗^ = highly significant.

**Table 4:** Correlation analysis between identified skin lesions and the onset of COVID-19.

Parameters		All Studied patients		Early		Late		Correlations		
		*N*	Percent (%)	*N*	Percent (%)	*N*	Percent (%)	*X* ^2^	*P* value	Significance
		150	100	66	44.0	84	56			

Maculo-papular rash	No	102	68.0	39	59.1	63	75.0	4.299	0.038	Significant
	Yes	48	32.0	27	40.9	21	25.0			

Vesiculobullous	No	91	60.7	36	54.5	55	65.5	1.851	0.174	Not significant
	Yes	59	39.3	30	45.5	29	34.5			

Urticarial lesions	No	114	76.0	54	81.8	60	71.4	0.121	0.079	Not significant
	Yes	36	24.0	12	18.2	24	28.6			

Cutaneous thromboembolic manifestations	No	105	70.0	66	100.0	39	46.4	50.51	0.001	Significant
	Yes	45	30.0	0	0.0	45	53.6			

EM like lesions	No	129	86.0	66	100.0	63	75.0	19.186	0.001	Significant
	Yes	21	14.0	0	0.0	21	25.0			

Rare cutaneous signs	No	129	86.0	57	86.4	72	85.7	0.013	0.909	Not significant
	Yes	21	14.0	9	13.6	12	14.3			

Note 3: EM: erythema multiforme; *X*^2^: chi-square test.

## Data Availability

All data generated or analyzed during this study are available from the corresponding author upon reasonable request.

## Supplementary Materials

Supplementary Materials Supplementary Figure 1: graph showing the demographic variables (sex and age) of patients involved in the current study.
