# Supplementary material for: Morphological and Prognostic Values of Skin Lesions in Patients with COVID-19
Source: Dermatol Res Pract. 2024 Oct 22;2024:4975523. doi: 10.1155/2024/4975523 (PMC11521579; doi:10.1155/2024/4975523)
Supplement: Supplementary Materials — Supplementary Figure 1: graph showing the demographic variables (sex and age) of patients involved in the current study. [file 4975523.f1.pdf]

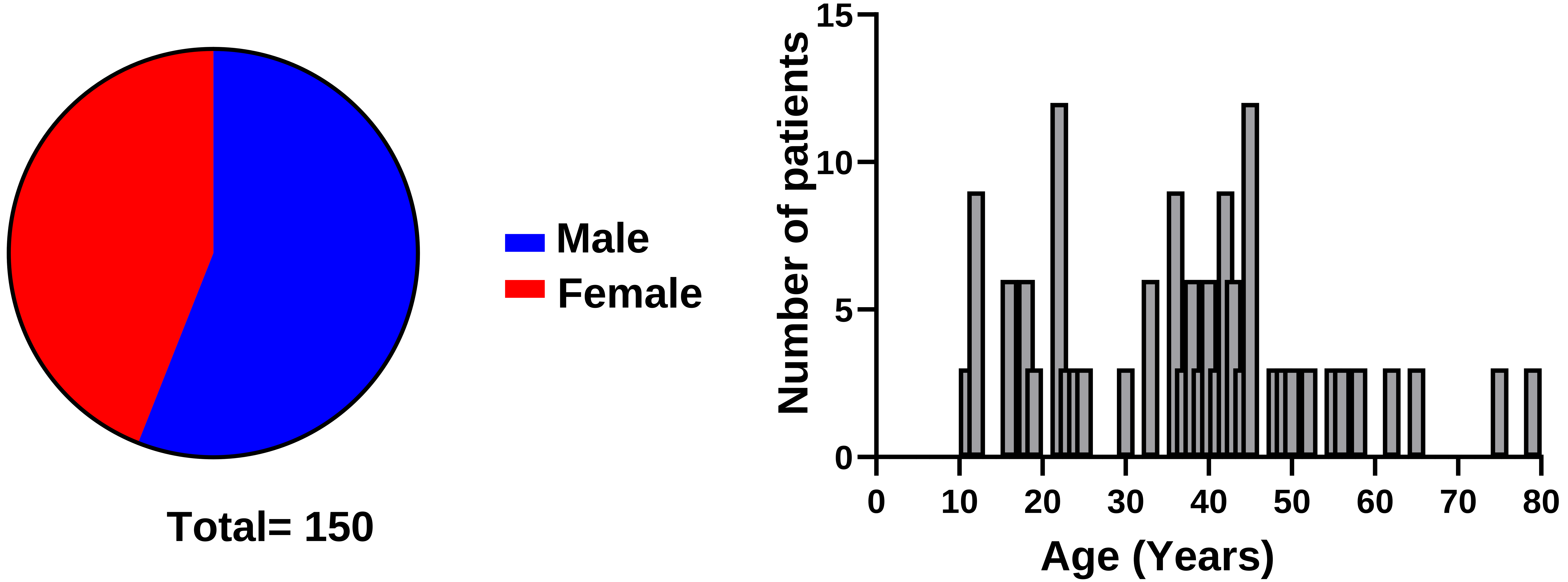

Supplementary Figure 1: Graph showing the demographic variables (sex and age) of patients involved in the current study.
